# Multidisciplinary Evaluation of Pacifier Removal on Oro-Dentofacial Structures: A Controlled Clinical Trial

**DOI:** 10.3389/fped.2021.703695

**Published:** 2021-09-13

**Authors:** Kelly Guedes de Oliveira Scudine, Camila Nobre de Freitas, Kizzy Silva Germano Nascimento de Moraes, Silvana Bommarito, Rosana de Fátima Possobon, Rosana Cristina Boni, Paula Midori Castelo

**Affiliations:** ^1^Department of Health Sciences and Pediatric Dentistry, Piracicaba Dental School, University of Campinas (UNICAMP), Piracicaba, Brazil; ^2^Department of Morphology, Piracicaba Dental School, University of Campinas (UNICAMP), Piracicaba, Brazil; ^3^Department of Speech Language Therapy, Federal University of São Paulo (UNIFESP), São Paulo, Brazil; ^4^Specialization Center in Speech Language Pathology (CEFAC), São Paulo, Brazil; ^5^Department of Pharmaceutical Sciences, Federal University of São Paulo (UNIFESP), Diadema, Brazil

**Keywords:** pacifier sucking, child development, bite force, speech, breathing, stomatognathic system

## Abstract

It is well recognized that pacifier habit leads to occlusal and orofacial functional changes in children. However, the effects of the interruption of prolonged pacifier habit on the development of the dento-facial complex has not yet been fully characterized. Thus, the aim of this study was to investigate the influence of pacifier removal on aspects of oro-dentofacial morphology and function in preschool children. For that, a pacifier group (*n* = 28) and a control group (*n* = 32) of 4-year-old children with and without pacifier habit, respectively, were followed up by a group of dentists and speech therapists at baseline, 6 and 12 months after habit removal. Bite force and lip pressure were assessed using digital systems, and the evaluation of breathing and speech functions was performed using validated protocols, together with the measurements of dental casts and facial anthropometry. The Two-way mixed model ANOVA was used in data analysis. After 12 months, a decrease in malocclusion frequency was observed in pacifier group. Additionally, a change over time was observed in facial, intermolar and palate depth measurements, as well in bite and lip forces and speech function scores, increasing in both groups (*p* < 0.01). The upper and lower intercanine widths and breathing scores differed between groups at baseline and changed over time reducing the differences. The presence of speech distortions was more frequent in the pacifier group at baseline and decreased over time (*p* < 0.05). The interruption of pacifier habit improved the maxillary and mandibular intercanine widths, as well as the breathing and speech functions, overcoming the oro-dentofacial changes found.

**Trial Registration:** This clinical trial was registered in the Brazilian Clinical Trials Registry (ReBEC; http://www.ensaiosclinicos.gov.br/), protocol no. RBR-728MJ2.

## Introduction

Non-nutritive sucking habits (NNSB), such as pacifier use and thumb-sucking, are generally engaged by infants in response to frustration and to satisfy their urge and need for contact (1). It has been suggested positive effects of the use of pacifier as a nonpharmacological intervention in the management of acutely painful procedures in infants (2, 3). Research has also demonstrated that pacifiers are associated with protection of sudden infant death syndrome (4). However, potential complications of non-nutritive sucking habits include early weaning, increase the likeliness of otitis media, malocclusion and undesirable dental arch traits at the end of the primary dentition (2, 5, 6). Many studies showed that persistence of these habits beyond 2 or 3 years of age considerably increases the probability of developing orthodontic problems, including anterior open bite, increased overjet, posterior crossbite and long facial height (7).

As form and function are closely related, incorrect orofacial functions have also been associated with malocclusion and NNSH in children (8). Prolonged duration of sucking habits produces harmful functional stimuli, which may jeopardize the position and strength of stomatognathic structures, with a detrimental impact on oral functions, including mastication, breathing and speech (9). The dental arches, acting as structural boundaries for placement of the tongue and lips, are intrinsically involved in sound production (10). Additionally, evidence suggest that sucking habits and mouth breathing are both closely related to anterior open bite, posterior crossbite and increased overjet (11).

While the negative consequences of prolonged NNSH on the primary dentition is well stablished in the literature (5, 6), scarce information about the effects of removal of sucking habits on orofacial functions has been documented. Until today, researchers focused on evaluating the oral myofunctional aspects in children with sucking habits using cross-sectional designs, which have many limitations and difficulties to provide evidence for a causal relationship (12, 13). One study evaluated some oral myofunctional structures in 36- to 60-month-old children, showing a higher prevalence of alterations in cheek mobility in pacifier users, considering both conventional and physiological ones, compared to habit-free children (12). Similarly, another survey showed that children with anterior open bite, with current or past pacifier sucking habit, presented higher prevalence of inadequate lip and tongue posture at rest and alteration of lip tonus (13). However, although myofunctional recovery is generally assumed by health professionals, it has not yet been proven whether there is a self-correction in orofacial functions after the pacifier is removed.

Therefore, the aim of this study was to evaluate prospectively the effects of pacifier removal on occlusal characteristics and orofacial functions in preschool children using a multidisciplinary approach. Our main hypothesis is that if stimulation from the pacifier use is removed, orofacial and dental structures will return to their normal and balanced growth.

## Materials and Methods

### Study Design

This is a controlled clinical trial with interventional and two-arm parallel design, registered in the Brazilian Clinical Trials Registry (ReBEC; http://www.ensaiosclinicos.gov.br/), protocol no. RBR-728MJ2. We followed the CONSORT guidelines in reporting this clinical trial.

Children born in 2013 and 2014 were tested in their usual kindergartens every 6 months from the year they turned four to the year they turned five. The children were recruited in 7 different kindergartens of the city of Piracicaba, São Paulo (Brazil). Evaluations were performed in three moments: at baseline, 6 months and 1 year of follow-up.

This study was approved by the Research Ethics Committee of the School of Dentistry of Piracicaba, University of Campinas, under Protocol No. 1.712.802. Written parental consent was requested before the beginning of the study and all procedures were performed in accordance to the Declaration of Helsinki.

### Sample

Children selected from seven public kindergartens in the municipality of Piracicaba, state of São Paulo (Brazil), composed the sample of this study. It is important to mention that the seven kindergartens were located in different neighborhoods of the municipality (downtown and suburb), chosen by the Educational Dept. of the municipality of Piracicaba. The number of participants to be included was based on the study of Verrastro et al. (2007) and considered the proportion of changes in lip posture and tonus found in children with normal occlusion and open bite (13). Considering a confidence level of 0.95 and power of 0.80, 32 participants in each group was needed.

After anamnesis and clinical examination, and based on inclusion and exclusion criteria, a total of 148 eligible children was selected (99 for the pacifier group and 49 for control group). From them, 47 children from pacifier group were excluded because parents were not willing to collaborate in removing the pacifier habit. Among the 52 participants who were willing to give up the habit, 24 children dropped out of the study because they moved from kindergarten or city during the year. In the control group, 13 participants dropped out of the study because they moved from kindergarten during the study and 4 children did not cooperate with the evaluations.

**Figure 1:**
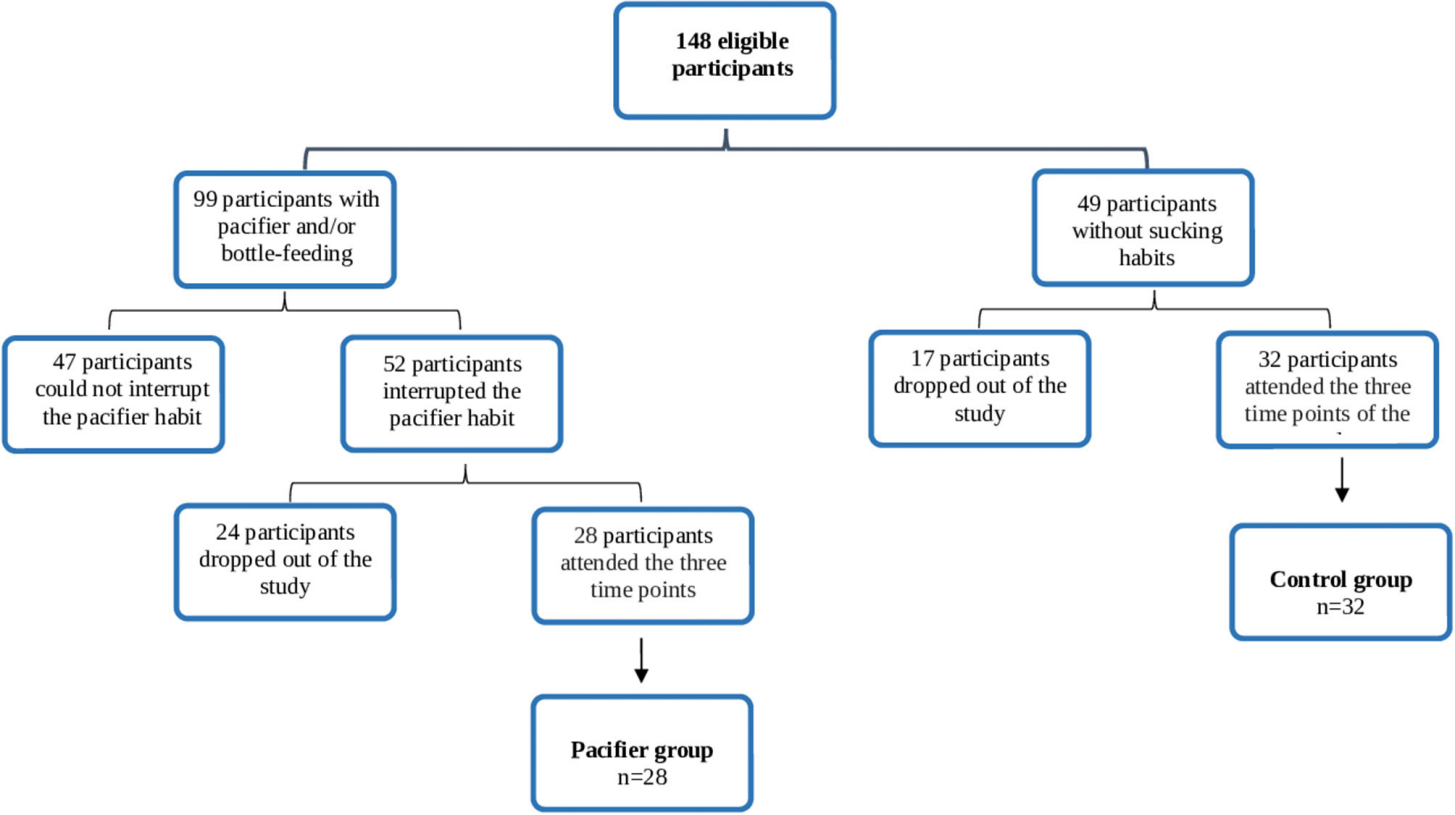
Flowchart depicting the number of participants throughout the study and the final sample in each group.

Thus, the final sample included 60 children who were divided into two groups: pacifier group consisting of 28 children with NNSH (pacifier) and nutritive sucking habits (bottle-feeding), and a control group with 32 children without any sucking habits and with normal occlusion. A flow chart shows the number of participants throughout the study and the sample which completed the sessions (Figure 1).

### Anamnesis

The child's caregiver was interviewed to obtain information regarding personal data, medical and dental history, nutritive and non-nutritive sucking habits during a structured personal interview. The exclusion criteria were as follows: presence of dental cavities, dental anomalies of number or shape and crossbite; history of oral or facial injuries; history or presence of orthodontic or speech therapy treatment; parents or caregivers' report or diagnosis of respiratory and/or food allergies; presence of systemic or local disorder that may compromise the masticatory system (e.g., neurological disorders, epilepsy, cerebral palsy, among others); use of medications that may directly or indirectly interfere with muscle activity, such as antihistamines, sedatives, syrups, homeopathy, or other central depressant drugs; patients who do not collaborate with data collection.

The inclusion criteria for the pacifier group were the presence of established deciduous dentition and NNSH (pacifier). Children with digit sucking were not included in this study. Considering the control group, current bottle-feeding and/or non-nutritive sucking habits were considered as exclusion criteria, and children with a history of NNSH was only admitted if that persisted up to 2 years of age. In addition, the control group should have normal occlusion.

### Method of Clarification and Positive Reinforcement for Removing the Pacifier Habit

The pacifier group was submitted to the method of clarification and positive reinforcement, which consists of clarifications about the possible clinical changes that the non-nutritive sucking habits (pacifier and bottle-feeding) could determine in the patient followed by strategies to reinforce the desired behavior (14, 15). Parents or guardians were aware of the importance of their integration in the context of the problem and, to elucidate this, photographs and pictures of books about the possible clinical changes that pacifier may produce in their children were shown. Children were also instructed that pacifier sucking could alter the position of their teeth and the guidance was conducted using both their own teeth (with mirrors) and pictures or models of other teeth with undesirable aesthetics. The second part of the strategy involved the parent rewarding the child for periods of “no sucking” by giving them their full attention and ignoring the sucking behavior if it occurred at any time of the day. The number of sessions focused on the orientation and awareness of children and their parents varied between 4 to 6 meetings. Illustrated calendars were also distributed for each day and night of one week, in which the child was instructed to draw pictures or markings when not using pacifier. The child was encouraged to make many drawings and thus could exchange them for stickers. With this instrument, it was possible to observe the frequency decrease until the habit elimination. Although the focus of the present study was the pacifier sucking habit, strong recommendations for the removal of bottle-feeding were also given to the parents during the meetings.

### Clinical Examination

One trained examiner, Dentist and Specialist in Orthodontics (KGOS), performed the clinical oral examination. The exam was carried out in a brightly lit kindergarten room to exclude participants with cavities and/or crossbite which are known to have an influence on masticatory parameters. The anthropometric assessment involved measurements of height and weight by an analogical scale and stadiometer to calculate the Z-scores for BMI according to World Health Organization (16).

Alginate impressions of the maxillary and mandibular arches and wax bite registrations were obtained from all children. All study casts were assessed by the same examiner. The presence of malocclusion was defined according to the criteria proposed by Baume (1950) and Foster & Hamilton (1969) (17, 18). Dental arch dimensions, including maxillary and mandibular intercanine widths and intermolar widths were recorded using a digital caliper (Mitutoyo, São Paulo, Brazil) as follows:

1. Maxillary and mandibular intercanine width: the distance (mm) between the most cervical lingual portion of the maxillary and mandibular right and left deciduous canine. The landmarks were placed at the gingival margin of the teeth on the assumption that the measurement is not affected by attrition or malposition of the teeth.
2. Maxillary and mandibular intermolar width: the distance (mm) between the central fossae of the right and left deciduous second molars in both arches.
3. Palatal depth: measured from the deepest point in the palate to a line connecting the mesiolingual tips of the deciduous second molars cusps.
4. Overjet and overbite measurements: overjet was measured as the distance between the palatal surfaces of the most projected maxillary incisor to the corresponding mandibular incisor and later classified as normal (≤ 2 mm) or increased (>2 mm). The overbite was considered to be normal when the upper incisors overlapped the lower incisors by 2 mm; a deep overbite was characterized by the maxillary teeth covering more than 2 mm of the vestibular surface of the mandibular teeth, and anterior open bite was considered to be the absence of a vertical overlap covering the lower incisors.

To reduce the effect of accidental errors and improve reliability, the mean of 3 consecutive measurements of 12 study casts, which were accepted only if they differed by less than 0.5 mm.

Facial morphology was evaluated using a sliding caliper (Bone Caliper in 240 mm aluminum, Cescorf, Brazil) according to the morphologic facial index, defined as the ratio between morphological facial height and the bizygomatic width (19). For each participant, the craniometric points were assessed by palpation/inspection and marked directly on the skin using an eyeliner. All participants were seated in a relaxed position, with the Frankfort plane horizontal to the floor and teeth in the intercuspal position.

Morphological facial height was defined as the distance between nasion and gnathion, and bizygomatic width as the distance between the zigion points. The distances nasion-gnathion and zigion-zigion were evaluated in millimeters, considering the following landmarks: the nasion is the most anterior point of the fronto-nasal outline in the midline; gnathion is the most anterior and inferior point of the bony chin; and zygion is the most lateral point on the zygomatic arch (7, 20).

### Maximal Bite Force

Maximal bite force was evaluated using a digital gnathodynamometer specially designed for this age group and (young children) (Dinamômetro Digital Kratos model DDK, Kratos Equipamentos Industriais Ltda., Cotia, Brazil), with fork strength of 10 mm connected to a digital device which provided the unilateral bite force in Newton (N) (20). The fork was placed bilaterally over the first primary molars, and the recordings were performed twice, with an interval of 1 min. During the test, subjects were seated in an upright position with the head in a natural position, keeping the Frankfort plane parallel to the floor. Before the recordings, each child was instructed to bite the fork as forceful as possible. The maximum value measured was defined as the maximum bite force. A pilot study was previously conducted in order to verify the reproducibility and to calculate the intraclass correlation coefficient (ICC) using the data of 15 children that were not part of the study sample. The intra-examiner reproducibility for bite force data (ICC = 0.97) was excellent.

### Assessment of Lip Strength

Lip strength was measured using the Iowa Oral Performance Instrument (IOPI system), model 2.2 (Northwest Co., LLC, Carnation, WA, USA). During the examination, the subjects remained seated in a comfortable chair with their feet flat on the floor and their head parallel to the horizontal plane. The IOPI system is formed by a pressure transducer that connects to a plastic bulb, which contains air inside. The device measures lip compression by measuring the maximum pressure peak exerted on the bulb, expressed in kilopascals (kPa). After instructions, the bulb was positioned between the lips in the midline and the child pressed it as hard as possible for two seconds (21). Each child was instructed to occlude their teeth to prevent the action of the jaw-lifting muscles. Maximum lip compression was measured three times with one-minute rest periods between measurements and the maximum generated pressure was considered as the final value. The intra-examiner reproducibility of lip strength values during the pilot study with 15 children was good (ICC = 0.77).

### Speech - Language Evaluation

The functions of breathing and speech were evaluated by means of different protocols, using recordings that were evaluated by one speech-language pathologist using blinded procedure. All the tests were recorded on video using a digital camera (Canon EOS Rebel T3I), at a standardized distance (1 m) from the subject, fixed on a tripod with focus on the face, neck and shoulders. During this evaluation, the child remained sitting in a chair with backrest and the feet resting on the floor.

Breathing mode was assessed using the Orofacial Myofunctional Evaluation with Scores-expanded (OMES-e) protocol, classifying the function as nasal or oronasal. The examiner attributed scores on a 4-point scale (the lower the score achieved, the more altered the function): 4 = when the lips remained in occlusion without effort, mainly during situations of rest and mastication, with the tongue contained in the oral cavity (normal pattern); 3 = mild alteration, when the subject presented oronasal inspiration but was able to perform inspiration only through the nose without showing signs of fatigue and dyspnea, 2 = moderate alteration when the condition was similar to the previous one but the subject did not maintain a nasal pattern, and 1 = severe alteration when the subject, while trying to perform nasal only inspiration, showed signs of fatigue and dyspnea and opened his mouth to inspire within a few seconds, a pattern observed both at rest and during the mastication of a cookie (22, 23). During the interview with the parents, the breathing mode was also verified.

Speech was assessed using the “Speech” domain of the “Orofacial Myofunctional Assessment - MBGR Protocol” (24). This protocol allows the speech-language pathologist to assess omission, substitution and distortion during picture naming, using for that a board of figures. Considering speech distortions, the following tongue aspects were assessed: “Anterior Interposition”, “Lateral Interposition”, “Absence or Reduction Vibration of Tip”, “Multiple Vibration of Tip”, “Elevation of Dorsum” and “Lowering of the Dorsum.” Spontaneous speech was also evaluated, asking the child the following questions: “tell me your name and age”, “tell me what do you like to play” and “tell me about a trip you enjoyed”, among other questions. In this protocol, the higher the score achieved, the more altered the function. The intra-examiner reproducibility of OMES-e (ICC = 0.86) and MBGR Protocol values (ICC = 0.70) during the pilot study with 15 children ranged from excellent to good.

### Statistical Analysis

Data were statistically analyzed using the SPSS 24.0 software (IBM Corp., NY, EUA) by one of the authors (PMC, Applied Statistics Spec), considering an alpha level of 5%.

Exploratory analysis consisted of means and standard deviation, medians and percentages. Normality was checked by using the Shapiro-Wilk test and Quantile-quantile-plot graphs (QQ-plot); non-normal distribution variables were transformed by using the natural logarithm, when necessary. Previously to analysis, duplicate or corrupted data, outliers, and missing data were identified. Categorical data were compared between groups and over time using Chi-squared/Fisher test and McNemar's test, respectively.

A general linear model – Two-way mixed model ANOVA - was used to test the effects of within-subjects factor (time: baseline, 6 months and 1 year) and the between-subjects factor (group: *control* and *pacifier*) and the interaction between these factors in the observed variance of the orofacial structure's morphology and functions (considered as dependent variables). The effect size (partial *Eta* squared) and the power of the test for each model were also obtained. The results of the Box's test, Mauchley's sphericity test and Levene's equality of variances were evaluated as assumptions; when necessary, the Huynh-Feldt correction was applied. Outliers were considered when the studentized residual was greater than ±3SD; no data imputation or elimination was needed/performed.

## Results

Considering the total sample of pacifier users included at the beginning of the study (*n* = 52), the intervention was effective to remove the habit in 28 children. Relative to the final sample of pacifier group (28 children), the average time to remove the sucking habit using the method of clarification and positive reinforcement was 1.9 months.

Table 1 shows the demographics and characteristics of sucking habits for both clinical groups at baseline. All children included in the pacifier group had a history of bottle-feeding, but the habit was present in 57% of children in the beginning of the study. Recommendations for the removal of bottle-feeding were also delivered to the parents during the meetings; however, 7% still remained with nutritive sucking habit after 1-year follow-up. Regarding the type of pacifier, parents reported that 84% of children used an “orthodontic” one. Only 20% of the children in the control group were exclusively breast-fed, and those who remained with the bottle-feeding habit stopped it before the study started.

**Table 1:** Demographic and clinical characteristics of groups at baseline.

**Group**	**n**	**Sex**	**Age** ** (months)**	**BMI** ** (Kg/m^**2**^)**	**Current pacifier use**			
					**Age of onset** ** (months)**	**Type of pacifier**	**Pacifier use during the day** ** (hours)**	**Pacifier use during the night** ** (hours)**
		**f/m**	**Mean** **(SD)**	**Mean** **(SD)**	**Median**	**%**	**Mean** **(SD)**	**Mean** **(SD)**
Control	32	16/16	48.2 (4.1)	15.7 (1.9)	–	–	–	–
Pacifier	28	15/13	48.2 (4.1)	15.8 (1.5)	12.0	orthodontic: 84 conventional: 16	3.9 (3.6)	8.9 (2.4)

*SD, standard deviation; BMI, body mass index; f, females; m, males*.

**Figure 2:**
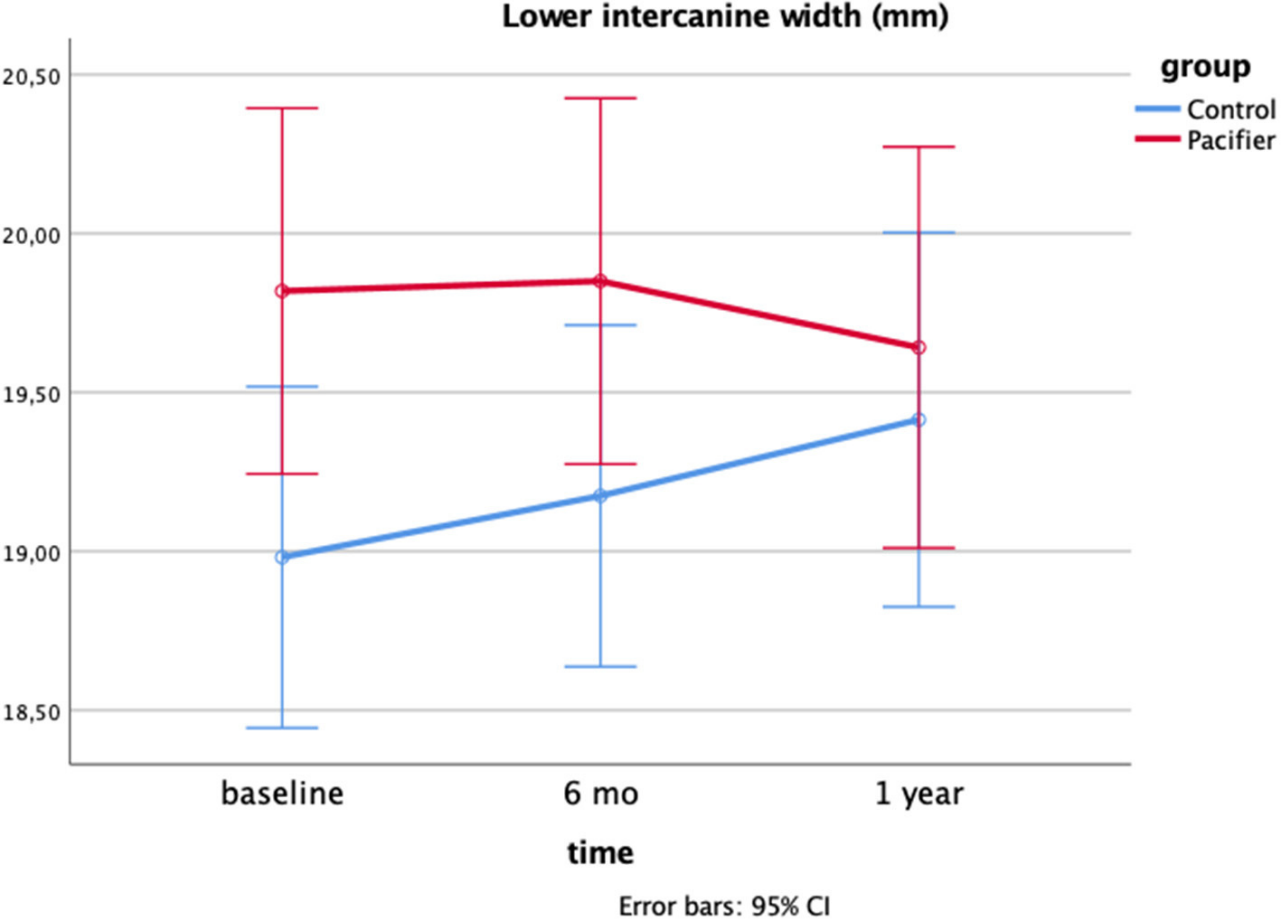
Interaction effect time*group on the lower intercanine width after 1-year follow-up: a two-way Mixed model (*p* = 0.020; *eta* partial squared = 0.07; power = 71%).

Table 2 shows the means (SD) of the morphological aspects of orofacial structures: facial morphology and maxillary and mandibular measurements. Changes in these measurements were observed in both groups as an effect of the time, with the exception of the upper intercanine width, which increased only in the pacifier group. Besides, the lower intercanine width differed between groups at baseline, and it only increased in the control group overtime in a way that the difference between groups was absent after 1-year of follow-up, as observed in Figure 2.

**Table 2:** Interaction effect time*group on orofacial morphological aspects: a Two-way Mixed Model.

**Group**	**Time**	**n-gn/zy-zy** ** ratio**	**Lower** ** intercanine width (mm)**	**Upper** ** intercanine width (mm)**	**Lower** ** intermolar width (mm)**	**Upper** ** intermolar width (mm)**	**Palate depth (mm)**
**Mean (SD)**							
Control	baseline	0.88 (0.06)	19.0^A^ (1.3)	24.6^A^ (1.4)	36.2 (1.5)	40.6 (1.5)	13.5 (1.4)
(*n* = 32)	6m	0.85 (0.05)	19.2 (1.3)	24.8 (1.5)	36.5 (1.7)	40.7 (2.1)	13.9 (1.4)
	1y	0.85 (0.05)	19.4 (1.6)	24.5 (1.7)	36.7 (1.7)	41.0 (1.8)	14.0 (1.1)
Pacifier	baseline	0.85 (0.05)	19.8^B^ (1.7)	23.2^B^ (1.7)	35.6 (1.7)	39.5 (1.7)	13.1 (1.4)
(*n* = 28)	6m	0.84 (0.04)	19.9 (1.7)	23.9 (1.6)	35.9 (1.6)	39.9 (1.9)	13.1 (1.3)
	1y	0.84 (0.04)	19.6 (1.7)	24.1 (1.7)	36.0 (1.9)	40.3 (2.1)	13.4 (1.7)
***p-value*** **(** ***eta*** **partial** ^**2**^ **/power of the test)**							
Time effect		** <0.001** (0.18/0.99)	0.457 (0.01/0.18)	** <0.001** (0.16/0.99)	** <0.001** (0.17/0.99)	** <0.001** (0.16/0.99)	**0.023** (0.07/0.70)
Interaction effect time*group		0.155 (0.03/0.39)	**0.020** (0.07/0.71)	** <0.001** (0.17/0.99)	0.864 (0.00/0.07)	0.343 (0.02/0.24)	0.415 (0.02/0.20)

*SD, standard deviation. A ≠ B in the same column (p < 0.05; One-way ANOVA). Significant values are highlighted in bold*.

In both groups, the proportion of facial height in relation to facial width decreased over time, while the upper and lower intermolar width and palate depth increased after 1 year of follow-up.

**Figure 3:**
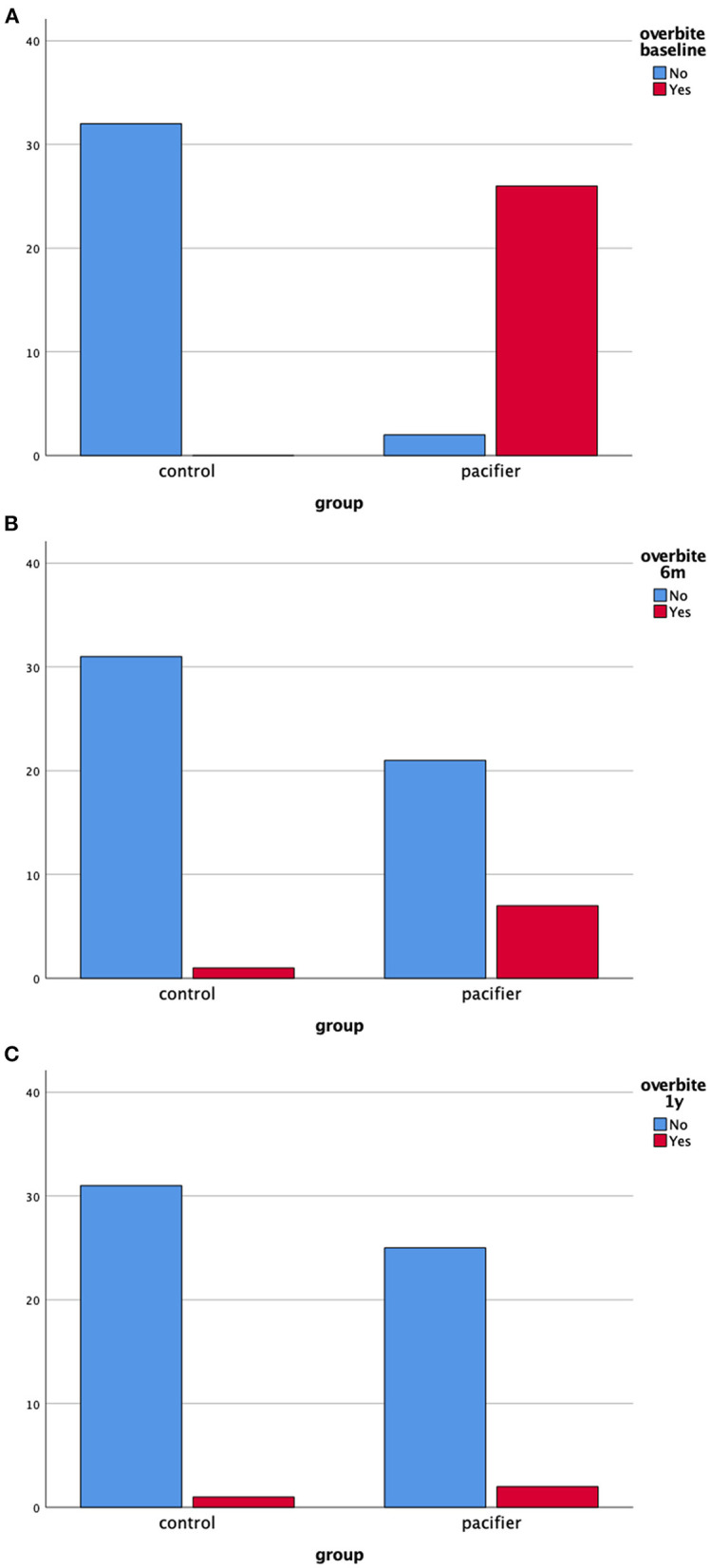
**(A–C)** Absolute frequency of overbite in the clinical groups at baseline, 6 months and 1-year follow-up (total *n* = 60).

Figures 3A–C show the frequency of overbite in both groups at baseline, 6 months and 1-year of follow-up, respectively. It is noteworthy the decrease in the absolute frequency of malocclusion 1-year after pacifier removal. The presence of open bite in the pacifier group was 85% at baseline (mean = 2.44 mm), 8% (1.5 mm) at the 6-month follow-up and 4% at the 1-year follow-up. Increased overjet was present in 68% of children who used a pacifier at baseline (mean = 4.35 mm), 42% at 1-year follow-up (mean = 3.5 mm) and 21% of children at 1-year follow-up (mean = 3.2 mm).

**Figure 4:**
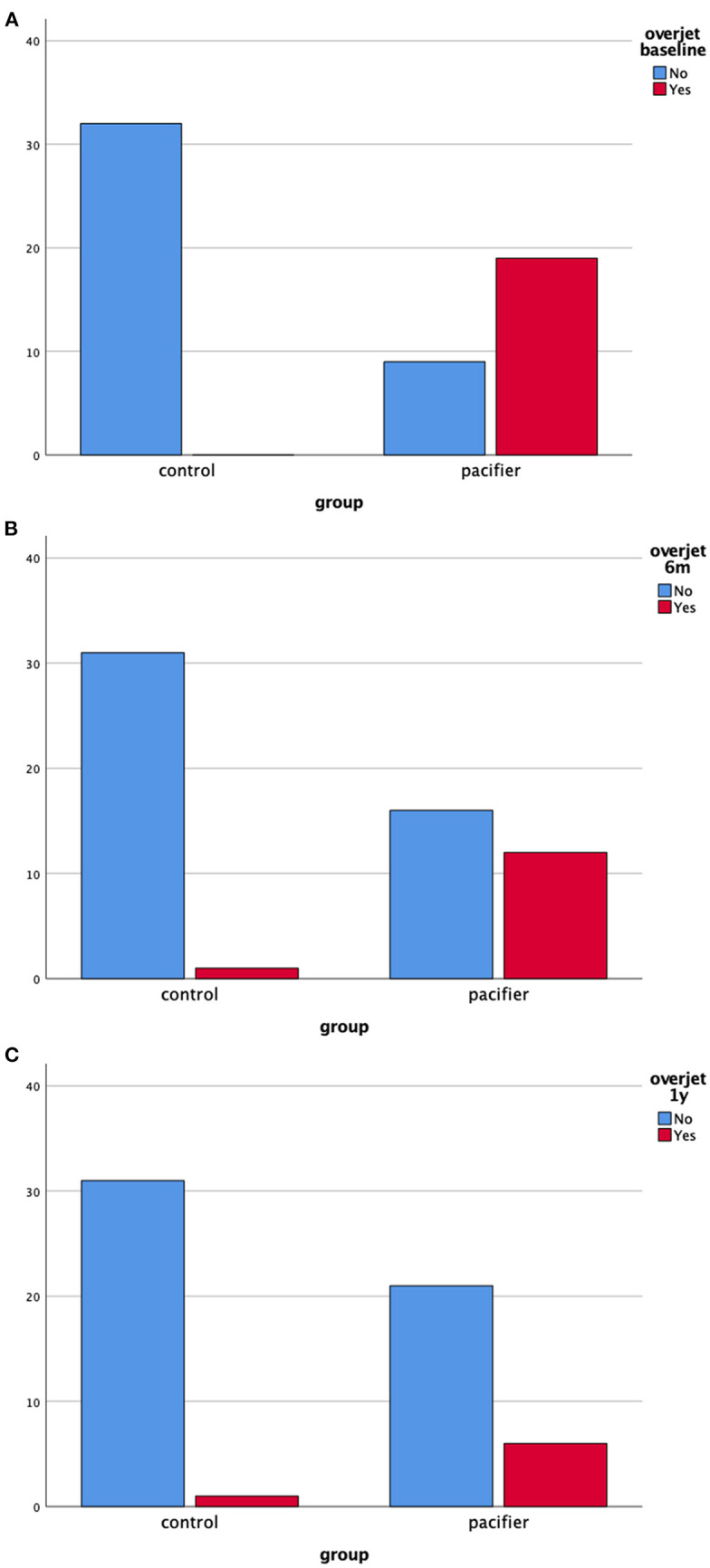
**(A–C)** Absolute frequency of overjet in the clinical groups at baseline, 6 months and 1-year follow-up (total *n* = 60).

Figures 4A–C show the frequency of overjet in both groups at baseline, 6 months and 1-year of follow-up, respectively. Again, it is of note the decrease in the absolute frequency of malocclusion 1-year after pacifier removal.

Table 3 shows the changes in functional aspects of orofacial structures: bite and lip forces, breathing and speech function scores. A change in bite force, lip force and speech function scores were observed in both groups as an effect of time, showing an improvement of these functions in both the control and pacifier groups.

**Table 3:** Interaction effect time*group on orofacial functional aspects: a Two-way Mixed Model.

**Group**	**Time**	**Bite force** ** left side** ** (N)**	**Bite force** ** right side** ** (N)**	**Lip force** ** (KPa)**	**OMES** ** Breathing score**	**MGBR** ** Speech score** ** (naming)**	**MGBR** ** Speech score** ** (spontaneous)**
**Mean (SD)**							
Control	baseline	258.2 (64.7)	249.3 (67.5)	4.6 (1.3)	3.6^A^ (0.6)	4.0 (1.8)	3.9 (1.9)
(*n* = 32)	6m	294.8 (61.4)	286.6 (59.5)	5.6 (1.7)	3.7 (0.5)	3.2 (1.9)	2.2 (2.2)
	1y	321.3 (58.3)	312.2 (58.2)	6.8 (2.0)	3.9 (0.4)	2.4 (1.9)	1.8 (2.0)
Pacifier	baseline	243.5 (67.2)	241.0 (63.9)	4.8 (1.5)	3.0^B^ (1.1)	4.8 (1.3)	3.7 (1.6)
(*n* = 28)	6m	272.9 (53.3)	272.3 (63.9)	5.9 (1.5)	3.5 (0.6)	3.2 (2.1)	2.0 (1.8)
	1y	267.7 (75.0)	282.5 (61.3)	7.7 (2.3)	3.8 (0.5)	2.2 (1.7)	1.5 (1.5)
***p-value*** **(** ***eta*** **partial** ^**2**^ **/power of the test)**							
Time effect		** <0.001** (0.20/0.99)	** <0.001** (0.29/1.00)	** <0.001** (0.36/1.00)	** <0.001** (0.24/1.00)	** <0.001** (0.42/1.00)	** <0.001** (0.43/1.00)
Interaction effect time*group		0.058 (0.05/0.56)	0.376 (0.02/0.22)	0.491 (0.01/0.17)	**0.023** (0.07/0.69)	0.143 (0.04/0.40)	0.988 (0.00/0.05)

*OMES, Orofacial Myofunctional Evaluation with scores; SD, standard deviation. A ≠ B in the same column (p < 0.05; One-way ANOVA). Significant values are highlighted in bold*.

**Figure 5:**
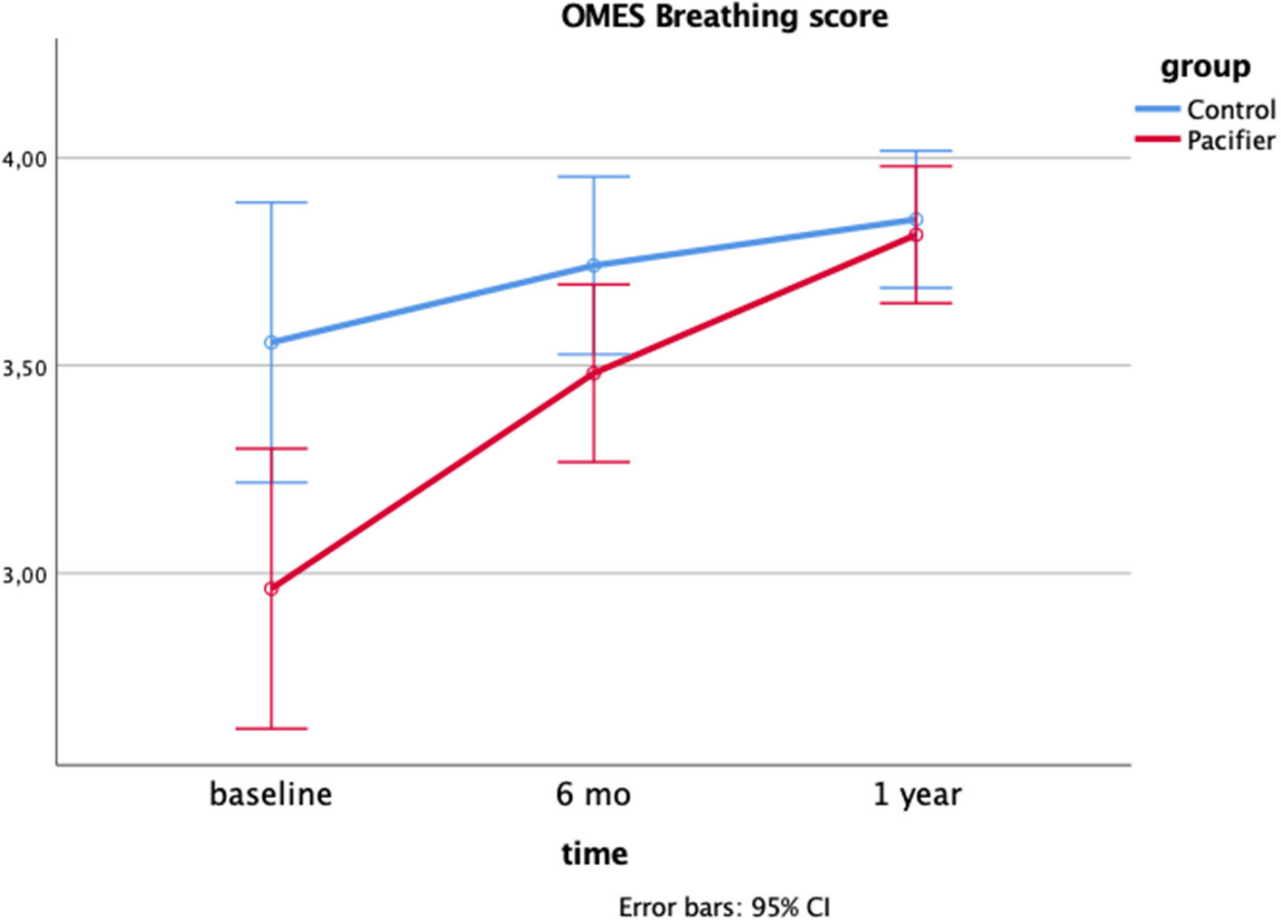
Interaction effect time*group on the OMES Breathing score after 1-year follow-up: a two-way Mixed model (*p* = 0.023; *eta* partial squared = 0.07; power = 69%).

For OMES breathing score, the change was dependent on the group: at baseline, scores were different between groups and a significant improvement was found in the pacifier group, as observed by the absence of difference at 1-year follow-up (Figure 5).

A closer look at the speech function showed that the frequencies of distortions related to tongue position between groups at baseline was different in the aspects “Anterior Interposition” (control group = 55% and pacifier group = 82%; *p* = 0.031) and “Absence or Reduction of Vibration of Tip” (control group = 6.5% and pacifier group = 50%; *p* = 0.016). Concerning the pacifier group, a significant decrease in the percentages of the distortion aspects “Anterior Interposition” and “Absence or Reduction of Vibration of Tip” were observed after 12 months of habit removal (*p* = 0.004). On the other hand, the distortion aspects ‘Multiple Vibration of Tip” and “Lowering of the Dorsum” were absent in both groups, and the aspects “Elevation of the Dorsum” and “Lateral Interposition” were observed in only two and three children of the pacifier group, respectively.

## Discussion

Although several studies have been conducted on pacifier use and its consequences, this is the first which prospectively evaluated the effects of removing the habit on occlusion and orofacial aspects. The data presented in this study confirm the association between prolonged pacifier use and alterations in dental arch dimensions and occlusion, as well as on speech and breathing functions, but now provides a comprehensive description of the improvement in maxillary and mandibular intercanine widths, together with the breathing and speech functions, after the interruption of pacifier habit, overcoming the oro-dentofacial changes found at baseline.

The use of a pacifier, as well as all other forms of NNSH, has been a focus of debate; in this regard, there is a consensus that its persistency for over than three years, usually produces significant occlusal disorders (6). In agreement with previous studies, the prevalence of malocclusion, mainly open bite, decreased significantly after removing the NNSH (25, 26). The open bite in pacifier-suckers occurs mainly due to a reduction in vertical growth in the anterior parts of the alveolar processes of the upper and lower jaws (27). In addition, with frequent and prolonged sucking, maxillary incisors are tipped buccally, whereas the mandibular incisors are tipped lingually, resulting in increased overjet. When the stimulus of the sucking habit is removed, incisal contact and a decrease in overjet is usually reached in a rather short time.

The method of clarification and positive reinforcement was successful in removing the habit of pacifiers in 54% of the sample. It is interesting to point out that there is no standard intervention for cessation of NNSHs. A variety of different approaches and interventions have been described in the literature, which range from fitting an orthodontic appliance to directly interfere with the habit, application of an aversive tasting substance to the pacifier or digit and psychological interventions (28). We recommend that this method be used by health professionals as a first attempt, as it has the advantages of being simple, inexpensive and not requiring visits to a dental office. Therefore, this study emphasizes the social and clinical importance of the method of clarification and positive reinforcement as an inexpensive and excellent strategy in public health to reduce the prevalence of sucking habits in early childhood.

Pacifier sucking habit was associated with a decreased maxillary intercanine width and increased mandibular intercanine width at baseline. The present findings are consistent with previous studies, in which prolonged NNSH were associated with changes in the intercanine measurements (29, 30)_._ The increased activity of the cheeks together with a reduced lingual support for the deciduous upper molars and canines, as the tongue is forced backward and downward by the pacifier nipple, contributes for the decreased maxillary intercanine width found. The lower position of the tongue may also favor to widen the lower arch and it is worth mentioning that these results were found even in a sample of children who did not present crossbite (28).

In the present study, palate depth increased after 1 year of follow-up in both groups. According to the literature, palatal depth increases continuously from the primary until the adult period, with a higher rate between 5 to 16 years of age (31, 32). Although we did not find differences between the groups regarding this measurement, a previous study showed that the pacifier habit was associated with palatal depth. Warren et al. investigated the association between the duration of NNSH and various occlusal characteristics in the primary dentition and found that palatal depths were significantly decreased among children with pacifier habits longer than 36 months (6).

Regarding facial morphology, no significant difference in morphological facial index was found between pacifier and control group. Our data corroborate a past study conducted in a group of children with similar age and ethnicity, in which facial morphology was not associated with the presence of sucking habits (7), highlighting the complexity of craniofacial growth and the importance of considering other etiological factors, such as nutrition, trauma and genetics (33). In fact, a population-based twin study showed that genetic factors can explain more than 70% of the phenotypic facial variation in facial size, nose, lips prominence and inter-ocular distance (34).

Evidence suggests that prolonged pacifier habit produce harmful functional stimuli, which may impair the position and strength of stomatognathic structures (9, 35). However, in the present study lip pressure and bite force did not differ between groups at baseline and they both increased over time, meaning that the NNSH did not have a significant impact on the magnitude of bite and lip forces at this age. A study of bite force in northern Japanese children showed that the magnitude may have significant variations concerning the different ages, in which nursery school children (3–5 years) displayed an average bite force of 186.2 N in males and 203.4 N in females (36). However, it would be interesting to follow the development of masticatory forces over a longer period of time. Regarding lip pressure, a previous study observed that lip pressure increases steadily from 5 months to 3 years, but slightly from 3 to 5 years, which corroborates our findings (37).

Breathing assessment was performed checking lip and tongue's posture, as well as the signs of fatigue and dyspnea during chewing and resting position. Ours results showed that children with pacifier sucking habit scored less in breathing evaluation and showed more oronasal breathing aspects before the intervention for the cessation of pacifier use. In oronasal breathing mode, lips are not sealed, the jaw is opened by the suprahyoid muscles and this displacement is followed by tongue (23). Similarly, a previous study observed that persistent non-nutritive sucking affected the prevalence of malocclusion and nasal breathing (38). Interestingly, a previous study demonstrated that myofunctional therapy reduced oral breathing, as well as restored normal tongue resting position in children with sleep-disordered breathing, highlighting the importance of a balanced growth and development of orofacial muscles for the correct development of breathing function (39). In addition, our data showed a self-correction after the interruption of the pacifier sucking habit, as observed by the absence of difference in the breathing scores at 1-year follow-up when compared to the control group. Although clinicians assume that this self-correction is an expected result, this is one of the few studies that investigated the changes in orofacial functioning resulting from the removal of prolonged pacifier use.

It is well known that the articulation of sounds depends on the position and mobility of the tongue, presence and position of the teeth (occlusion) and mobility of the lips and cheeks, which altogether promotes adequate intraoral space for phonemic articulation and resonance (40). In the present study, children from the pacifier group showed similar results in total score of the speech assessment through MBGR protocol when compared to the control group at all times evaluated. However, a closer look at the distortion aspects showed a higher frequency of changes in tongue posture in children with pacifier habit at baseline, including anterior tongue interposition and reduction of tongue tip vibration. As most children with a pacifier habit presented an open bite, the interposition of the tongue during speech is considered an adaptation, since the contact on the palatal surface of the upper incisors is not feasible (34). Yet, the reduction of tongue tip vibration during speech can be explained by a greater abnormal mobility of the tongue in children with prolonged sucking habits (41).

The current evidence around the relationship between NNSH and speech sound development is very limited and provides controversial findings (42). A study conducted in Australian English-speaking preschoolers showed that phonological impairment was not associated with a history of NNSH (43), while a three-fold increase in the relative odds of speech disorder was found in pacifier users for 3 or more years in a study with Patagonian preschoolers (44), corroborating the present results. However, it is important to note the wide variety of protocols that have been employed in the literature, and emphasize the strengths of the present findings, such as the use of a validated protocol in a controlled study design, contributing to the reliability of the results found.

It is important to note that children from the control group had significantly less occurrence and degree of changes in occlusion and oral myofunctional structures than their counterparts. In the present study, we also described normative data of myofunctional development of young children without sucking habits; this information is scarce in the literature and may serve as reference data that help health professionals planning and assessing myofunctional treatment outcomes in the pediatric population. Furthermore, the development of orofacial functions in a group of children “habit free” may be useful when considering different populations and conditions.

This study has some limitations and strengths that should be mentioned. Performing research in preschoolers is challenging due to the non-collaborative behavior in some procedures. First, we chose to exclude from the assessments the children who did not interrupt the pacifier habit for some reasons. The most important was ethical: we continued to offer counseling to all children and their parents in order to interrupt the habit, as there is sufficient evidence in the literature about the harmful effects of the habit's persistence. In addition, the duration of sucking habit would be different among these children, and a heterogeneous group of children with persistent habit would not be comparable and desirable. Another potential limitation was the losses that occurred during the follow-up period, which is relatively common in longitudinal designs; on the other hand, most of the differences found reached a sufficient power, strengthen our findings. Additionally, we should emphasize the multidisciplinary approach and the rigor of the methodology employed, such as the use of validated measures and the blinded procedure.

## Conclusion

The interruption of the habit improved the maxillary and mandibular intercanine widths, as well as the breathing and speech functions, overcoming the oro-dentofacial changes found in children with pacifier habit compared to control ones. Thus, the use of pacifiers should be discontinued as soon as possible, as their use can affect the occlusion and orofacial growth and development.

## Data Availability Statement

The raw data supporting the conclusions of this article will be made available by the authors, without undue reservation.

## Ethics Statement

The studies involving human participants were reviewed and approved by Research Ethics Committee of the School of Dentistry of Piracicaba, University of Campinas, under Protocol No. 1.712.802. Written informed consent to participate in this study was provided by the participants' legal guardian/next of kin.

## Author Contributions

KS contributed to the conception and design of the study, data collection, and article writing process. CF participated in data collection and data analysis. KN analyzed the videos to evaluate speech and breathing function. RP revised the manuscript critically for important intellectual content. RB participated in the analysis the data. PC contributed to the conception and design of the study, general supervision, and was responsible for the statistical analysis of the data. All authors have approved the final article.

## Funding

This work was supported by the State of São Paulo Research Foundation (FAPESP, SP, Brazil, grant number 2016/13867-0).

## Conflict of Interest

The authors declare that the research was conducted in the absence of any commercial or financial relationships that could be construed as a potential conflict of interest.

## Publisher's Note

All claims expressed in this article are solely those of the authors and do not necessarily represent those of their affiliated organizations, or those of the publisher, the editors and the reviewers. Any product that may be evaluated in this article, or claim that may be made by its manufacturer, is not guaranteed or endorsed by the publisher.
